# Nothing in SARS-CoV-2 makes sense except in the light of RNA modification?

**DOI:** 10.2217/fvl-2022-0043

**Published:** 2022-07-19

**Authors:** Mingmei Zhao, Chunxiao Li, Yu Dong, Xuekun Wang, Wenqing Jiang, Yaogang Chen

**Affiliations:** ^1^Department of Neurosurgery, Qingdao Center Hospital, Qingdao, Shandong, 266042, China; ^2^Cardiovasology Department I, Qingdao Center Hospital, Qingdao, Shandong, 266042, China; ^3^Interventional Catheterization Lab, Qingdao Center Hospital, Qingdao, Shandong, 266042, China; ^4^Department of Respiratory Diseases, The Affiliated Qingdao Hiser Hospital of Qingdao University, Qingdao, Shandong, 266033, China; ^5^Department of Respiratory Diseases, Qingdao Haici Hospital, Qingdao, Shandong, 266033, China

**Keywords:** ADAR, APOBEC, expression pattern, RNA deamination, SARS-CoV-2

## Abstract

The expression pattern of RNA deaminases determines the mutation and evolution of SARS-CoV-2.

COVID-19 mediated by SARS-CoV-2 is still causing damage to the world. One of the most challenging issues is the continuous mutation and evolution of the SARS-CoV-2 sequence. As we know, viral mutations may alter the pathogenesis, virulence and vaccine resistance of the virus, complicating the prevention and diagnosis of the disease. Therefore, understanding the molecular mechanism underlying the virus evolution is necessary.

In this article, we will propose our perspective that when studying the mutation and evolution of SARS-CoV-2, one would inevitably come to the topic of RNA deamination systems in hosts. Without considering RNA deamination, a study on SARS-CoV-2 would hardly make sense [1]. In memory of the evolutionary biologist Theodosius Dobzhansky [2], we claim that virtually nothing in SARS-CoV-2 makes sense except in the light RNA deamination.

## Evidence of virus evolution driven by deamination systems of hosts

Despite the fact that mutation of SARS-CoV-2 is one of the most worrying news during the pandemic, we should always be aware that the mutation of this ssRNA-positive virus must take place in cellular systems. One of the mutation mechanisms is the replication errors, where the mutations should: occur under a rate of 1E–8; and exhibit a symmetric distribution of all types of nucleotide substitutions [3,4].

However, in reality, the mutation profile in SARS-CoV-2 (both fixed and polymorphic sites) are obviously skewed to the C-to-U(T) substitution [5–7], and the occurrence of mutations is apparently higher than 1E–8 [8]. These facts indicate that: there are other mutation sources other than replication errors; and that traditional evolutionary algorithm/formula should not be directly applied to the SARS-CoV-2 evolution due to the different nature of mutation sources [9,10].

Fortunately, researchers have found that the C-to-U deamination by APOBECs [5,11] and the A-to-I deamination by adenosine deaminases acting on RNA (ADARs) [3] could nicely explain the most prevalent C-to-U substitution and the second-most prevalent A-to-G substitution in the SARS-CoV-2 sequence. The occurrence of deamination events is much higher than 1E–8 and it would produce an asymmetric distribution in the mutation profile [8]. Therefore, any studies dealing with the mutation and evolution of SARS-CoV-2 (no matter what the original purpose was) are actually dealing with the evolution patterns of RNA deamination sites. This is why nothing in SARS-CoV-2 makes sense except in the light RNA deamination.

## Expression pattern of RNA deaminases explains the mutation profile of SARS-CoV-2

To comprehend the evolution process behind the mutation profile of SARS-CoV-2, one should first understand how the RNA deaminases (ADARs and APOBECs) work.

(1) Tissue specificity: ADAR family has three members in humans (ADAR1–3) and all of them have considerable expressions in lungs and nerve systems. APOBECs are encoded by multiple subunits and the expressions of which are also enriched in lungs and nerves [12]. This suggests that when SARS-CoV-2 infects human lungs, it will be highly ‘mutable’ in lungs due to the tissue-specific expression of RNA deaminases.

(2) Subcellular localization: ADAR2 and ADAR3 are located in nucleus while ADAR1 only has partial expression in cytosol (the p150 isoform) [13]. In contrast, APOBECs have a much wider expression spectrum ranging from nucleus to cytosol as well as other cellular compartments. Since SARS-CoV-2 mainly invades the cytosol, the localization of deaminases successfully explains why C-to-U sites are more prevalent than A-to-I sites in SARS-CoV-2 (although these two deamination types are already much more prevalent than other substitutions).

(3) Sequence preference: ADAR3 is inactive in mammals, ADAR2 preferentially deaminates coding sequences and ADAR1 mainly deaminates noncoding regions [14]. Since most of the SARS-CoV-2 ‘genome’ is coding region, it is intuitive to think that ADAR2 should be the ‘chief editor’ of A-to-I events in SARS-CoV-2. Unfortunately, the nucleus-located ADAR2 is generally inaccessible to SARS-CoV-2. In contrast, the widely expressed APOBECs do not have the preference on coding/noncoding regions so that the C-to-U sites are catalyzed in an unbiased manner. This dilemma for ADARs again explains why A-to-I substitutions are much fewer than C-to-U substitutions in SARS-CoV-2.

Altogether, the expression patterns of RNA deaminases explain the mutation profile of SARS-CoV-2, and there is no reason to omit this information when one studies the sequence evolution of SARS-CoV-2.

## SARS-CoV-2 has the potential to infect human nerve systems, the tissues with abundant RNA deaminases

We have introduced that the expressions of ADARs and APOBECs are highest in lungs and nerve systems. Interestingly, apart from the commonly known infection in lungs, many cases suggest that SARS-CoV-2 could also infect nerve systems, leading to neurological symptoms like encephalopathy and delirium [15,16]. This scenario implies that SARS-CoV-2 seems to selectively (or ‘deliberately’) infect the tissues with high expression of RNA deaminases, accelerating its own mutation and evolution rate. However, we emphasize that tissue tropism is a separate problem that must not be related to the presence of host deaminases. The observation of SARS-CoV-2 infecting nerve systems might simply be the result of natural selection. One possible evolutionary trajectory is, among all the tissues or cell types, only lungs and nerve systems could force the virus sequence to change rapidly, providing more options for the virus to increase its fitness in hosts. Consequently, only the SARS-CoV-2 strains with adaptive mutations (which are randomly obtained) could survive. Therefore, the selectively maintained viral strains inherited the ability to infect nerve systems.

Without considering the RNA deaminases, one could hardly understand why SARS-CoV-2 infects lungs and nerve systems. Even with the structural evidence that only particular receptors on cell membrane could interact with SARS-CoV-2, this antigen–receptor relationship is highly susceptible to mutations. Natural selection is the only force to create a new host–virus relationship (by positive selection) or to maintain an existing host–virus relationship (by purifying selection).

## Parameters like linkage disequilibrium are only meaningful at single molecule level instead of individual host level

Among the numerous literatures on SARS-CoV-2 evolution, one could usually see some traditional analyses on linkage disequilibrium (LD), recombination, Theta-Pi or Tajima’s D [17]. Here, in the light of RNA deaminases, we would point out the flaws and paradoxes behind these previous studies.

For humans, each individual is a diploid so that the LD analysis could be performed among a human population. But for SARS-CoV-2, if one intends to perform LD, then how to define ‘a virus individual’ or a ‘haplotype’? The millions of viral sequences isolated from a single host (namely a sequencing library) would be highly polymorphic due to promiscuous deamination by ADARs and APOBECs (a phenomenon termed ‘intra-host polymorphisms’). In theory, each RNA molecule should be regarded as ‘a virus individual’ and the unit of LD should be each ‘single sequencing read’. Unfortunately, in most SARS-CoV-2 literature dealing with ‘strains’ [18,19], one sequencing library from one host is regarded as ‘one strain’ to perform LD [17] regardless of the intra-host polymorphisms caused by deamination. Moreover, misdefinition of ‘a virus individual’ also affects the definition of population size *N_e_*. It remains debatable whether the *N_e_* of SARS-CoV-2 refers to the number of infects humans, infected cells, or the number of SARS-CoV-2 RNA molecules. When RNA deamination and intra-host polymorphisms are considered, it becomes evident that each SARS-CoV-2 molecule should be treated as a virus individual. Likewise, evolutionary analyses on recombination, nucleotide diversity (Theta-Pi), selection strength (Tajima’s D) and allele frequency should also take the deamination events into account [20].

## Future perspective

In this article, we have introduced that the expression patterns of human RNA deaminases determine the: tissue-specificity of SARS-CoV-2 infection; the abundance of mutations; and the fast evolution of virus. We are also concerned that the previous definition of ‘a SARS-CoV-2 strain/haplotype’ should be updated due to the high level of intra-host polymorphism.

We appeal that the next-generation sequencing of SARS-CoV-2 isolates needs longer reads that cover the whole RNA in order to determine the linkage of distantly located mutation sites. The allele frequency and diversity analyses should be performed at single molecule level. Moreover, different from the human population genetics, the prevalence of mutations in SARS-CoV-2 is dictated by an additional factor, that is the activity of deaminases in host. Therefore, researchers should seriously consider what the mutation spectrum tells us before casually making a conclusion based on the incomplete observation.

## References

[1] Liu X, Liu X, Zhou J, Dong Y, Jiang W, Jiang W. Rampant C-to-U deamination accounts for the intrinsically high mutation rate in SARS-CoV-2 spike gene. RNA 28(7), 917–926 (2022). 35508354 10.1261/rna.079160.122 PMC9202584

[2] Dobzhansky T. Nothing in biology makes sense except in light of evolution. Am. Biol. Teach. 35(3), 125–129 (1973).

[3] Di Giorgio S, Martignano F, Torcia MG, Mattiuz G, Conticello SG. Evidence for host-dependent RNA editing in the transcriptome of SARS-CoV-2. Sci. Adv. 6(25), eabb5813 (2020). 32596474 10.1126/sciadv.abb5813 PMC7299625

[4] Zong J, Zhang Y, Guo F Poor evidence for host-dependent regular RNA editing in the transcriptome of SARS-CoV-2. J. Appl. Genet. 63(2), 413–421 (2022). 35179717 10.1007/s13353-022-00687-y PMC8854479

[5] Li Y, Yang X, Wang N Mutation profile of over 4500 SARS-CoV-2 isolations reveals prevalent cytosine-to-uridine deamination on viral RNAs. Future Microbiol. 15, 1343–1352 (2020). 33085541 10.2217/fmb-2020-0149

[6] Li Y, Yang XN, Wang N The divergence between SARS-CoV-2 and RaTG13 might be overestimated due to the extensive RNA modification. Future Virol. 15(6), 341–347 (2020).

[7] Li Y, Yang X, Wang N GC usage of SARS-CoV-2 genes might adapt to the environment of human lung expressed genes. Mol. Genet. Genomics 295(6), 1537–1546 (2020). 32888056 10.1007/s00438-020-01719-0 PMC7473593

[8] Zhang YP, Jiang W, Li Y Fast evolution of SARS-CoV-2 driven by deamination systems in hosts. Future Virol. 16(9), 587–590 (2021). 34721652 10.2217/fvl-2021-0181 PMC8547201

[9] Li Y, Yang XN, Wang N Pros and cons of the application of evolutionary theories to the evolution of SARS-CoV-2. Future Virol. 15(6), 369–372 (2020).

[10] Wei L. Reconciling the debate on deamination on viral RNA. J. Appl. Genet. (2022) (Epub ahead of print). 10.1007/s13353-022-00698-9 PMC9065659 35507138

[11] Harris RS, Dudley JP. APOBECs and virus restriction. Virology 479–480, 131–145 (2015). 10.1016/j.virol.2015.03.012 PMC4424171 25818029

[12] Martignano F, Di Giorgio S, Mattiuz G, Conticello SG. Commentary on “Poor evidence for host-dependent regular RNA editing in the transcriptome of SARS-CoV-2”. J. Appl. Genet. 63(2), 423–428 (2022). 35279801 10.1007/s13353-022-00688-x PMC8917825

[13] Patterson JB, Samuel CE. Expression and regulation by interferon of a double-stranded-RNA-specific adenosine deaminase from human cells: evidence for two forms of the deaminase. Mol. Cell. Biol. 15(10), 5376–5388 (1995). 7565688 10.1128/mcb.15.10.5376 PMC230787

[14] Tan MH, Li Q, Shanmugam R Dynamic landscape and regulation of RNA editing in mammals. Nature 550(7675), 249–254 (2017). 29022589 10.1038/nature24041 PMC5723435

[15] Helms J, Kremer S, Merdji H Neurologic features in severe SARS-CoV-2 infection. N. Engl. J. Med. 382(23), 2268–2270 (2020). 32294339 10.1056/NEJMc2008597 PMC7179967

[16] Liotta EM, Batra A, Clark JR Frequent neurologic manifestations and encephalopathy-associated morbidity in COVID-19 patients. Ann. Clin. Transl. Neurol. 7(11), 2221–2230 (2020). 33016619 10.1002/acn3.51210 PMC7664279

[17] Haddad D, John SE, Mohammad A SARS-CoV-2: possible recombination and emergence of potentially more virulent strains. PLoS ONE 16(5), e0251368 (2021). 34033650 10.1371/journal.pone.0251368 PMC8148317

[18] Shu YL, Mccauley J. GISAID: global initiative on sharing all influenza data – from vision to reality. Eurosurveillance 22(13), 2–4 (2017). 10.2807/1560-7917.ES.2017.22.13.30494 PMC5388101 28382917

[19] Zhu L, Wang Q, Zhang W, Hu H, Xu K. Evidence for selection on SARS-CoV-2 RNA translation revealed by the evolutionary dynamics of mutations in UTRs and CDSs. RNA Biol. 19(1), 866–876 (2022). 35762570 10.1080/15476286.2022.2092351 PMC9584556

[20] Yu YY, Li Y, Dong Y, Wang XK, Li CX, Jiang WQ. Natural selection on synonymous mutations in SARS-CoV-2 and the impact on estimating divergence time. Future Virol. 16(7), 447–450 (2021).

